# Supplementary material for: Acoustic probing of new biomarkers for rapid sickle cell disease screening
Source: Lab Chip. 2025 Dec 12;26(7):2187–200. doi: 10.1039/d4lc00847b (PMC12728720; doi:10.1039/d4lc00847b)
Supplement: LC-026-D4LC00847B-s003 [file LC-026-D4LC00847B-s003.pdf]

Supplementary Information (SI) for Lab on a Chip.
This journal is © The Royal Society of Chemistry 2025

Supplementary Information for

Acoustic Probing of New Biomarkers for Rapid Sickle Cell Disease Diagnosis

Nakul Sridhar[a], Meiou Song[a], Michael H.B. Stowell[b], Kathryn L. Hassell[c], Xiaoyun Ding*[a,d,e]

[a]Paul M. Rady Department of Mechanical Engineering, University of Colorado, Boulder, CO, USA.
[b]Department of Molecular, Cellular and Developmental Biology, University of Colorado, Boulder, CO, USA.
[c]Colorado Sickle Cell Treatment and Research Center, School of Medicine, Anschutz Medical Campus, University of Colorado, Aurora, Colorado, USA.
[d]Biomedical Engineering Program, University of Colorado, Boulder, CO, USA.
[e]BioFrontiers Institute, University of Colorado, Boulder, CO, USA.

*Corresponding author. Email: xiaoyun.ding@colorado.edu

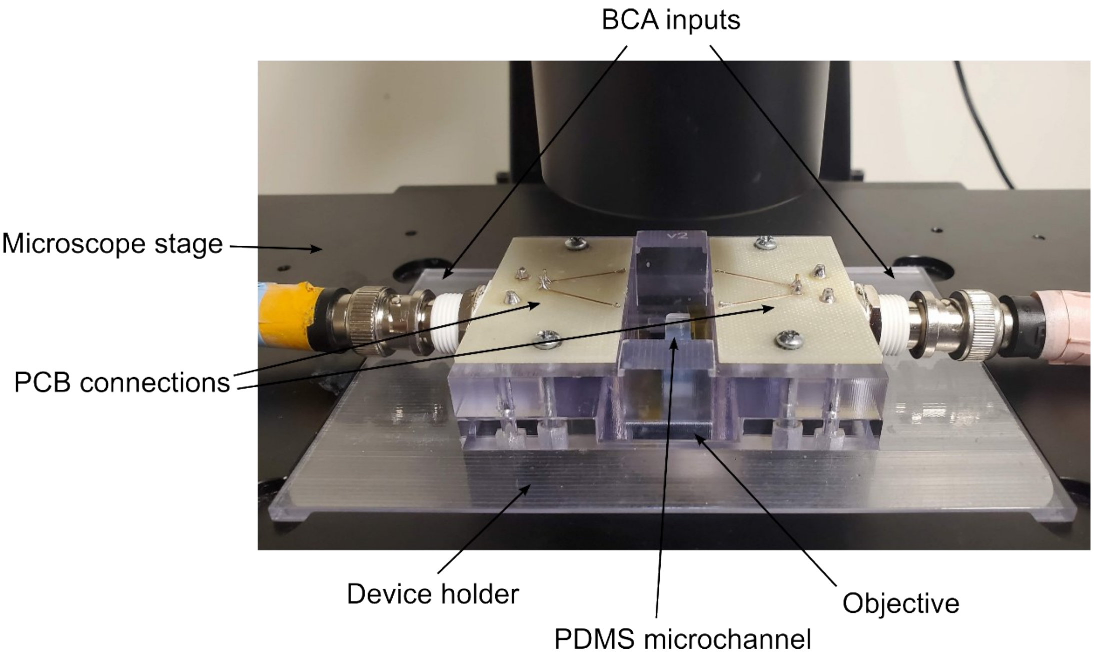

Figure S1. Experimental setup of acoustic probing device. The SAW chip is placed in a custom 3D printed holder fitted to the inverted microscope stage. Radio frequency (RF) signal is input via BCA cables to PCB connectors that transfer power to IDTs.

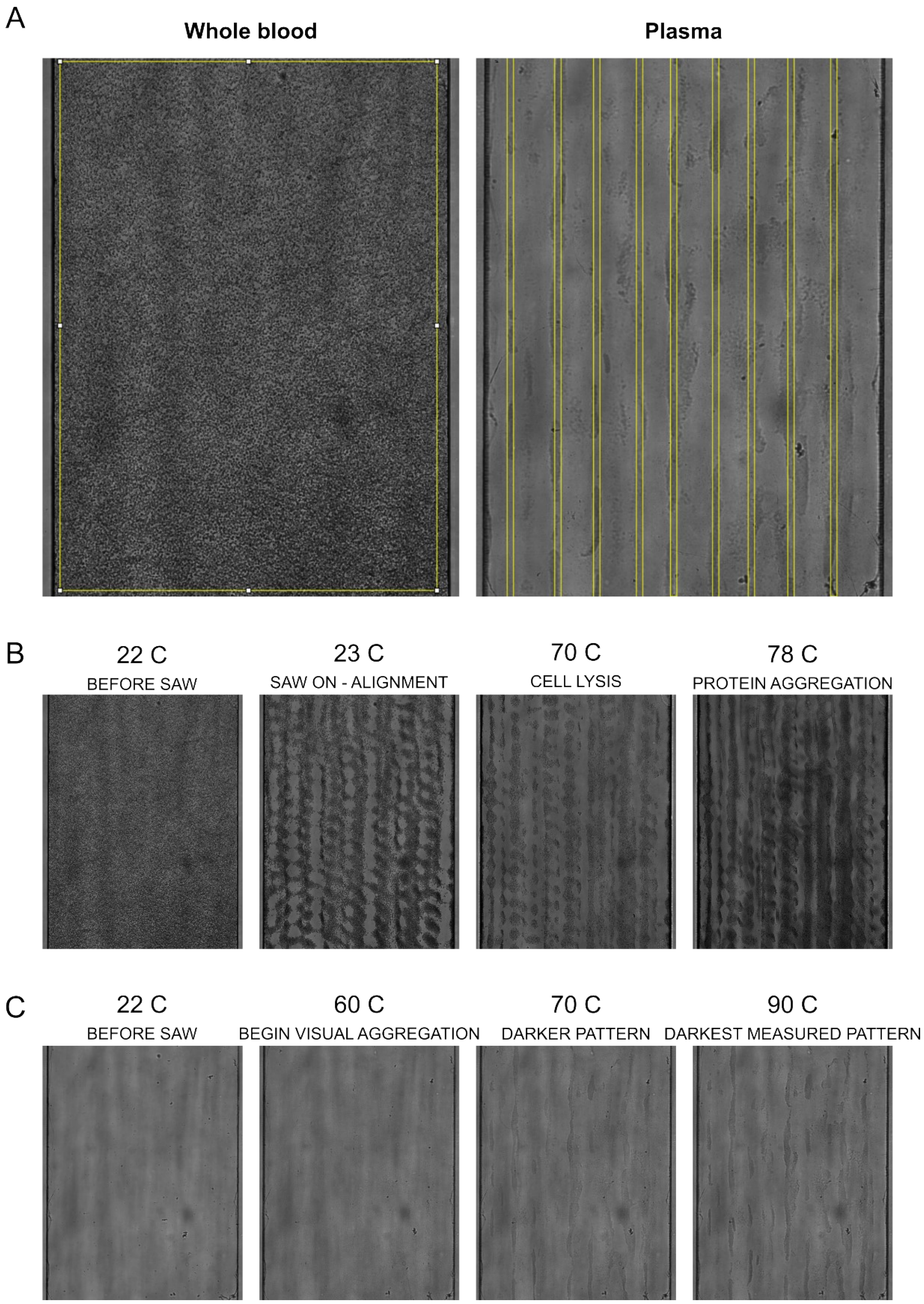

Figure S2. Unaltered raw images used for analysis. (A) Selected regions of interest (ROIs) (yellow) for whole blood and plasma diagnostic. Whole blood had stronger signal so the entire channel width could be used as ROI. Plasma produced an overall weaker signal, so the ROI was focused to the pressure nodal lines where proteins aggregated and the signal was strongest. **(B)** Raw representative images demonstrating cell alignment to pressure nodes, lysis, and protein aggregation in whole blood samples. **(C)** Raw representative images demonstrating gradual darkening due to increased protein precipitation and aggregation in plasma samples.

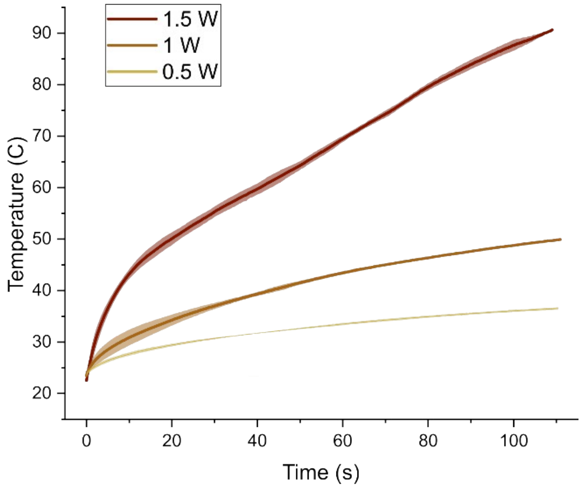

Figure S3. Characterization of acoustic heating rate. Various input powers were tested showing precise controllability of temperature in the channel. Data are means ± s.d. At least 3 independent trials were conducted for each condition.

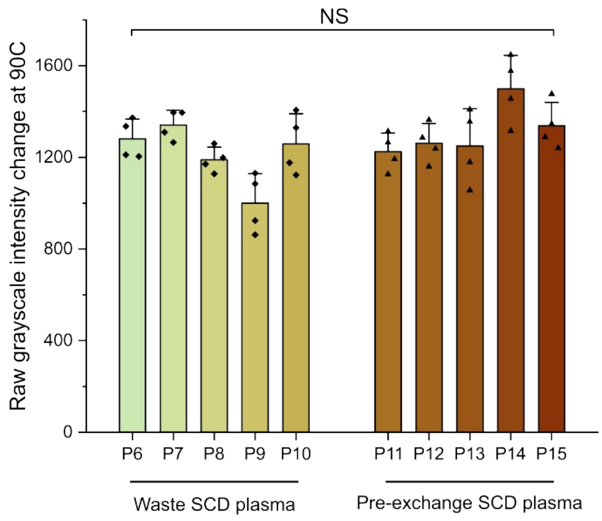

Figure S4. Comparison of relative plasma protein precipitation by calculating the average raw grayscale intensity difference measured at 90 C for individual donors taken from post-exchange waste HbSS donors and pre-/no exchange HbSS donors. Plasma is not replaced as part of the RBC transfusion exchange procedure, so there is no functional difference between the two groups. Data are means ± s.d. For each group, 5 individual donors were tested with at least 4 independent trials per donor. For statistical analysis, data were aggregated per donor to performing a two-sided, unpaired Student's t-test. Waste HbSS vs. Pre-exchange HbSS: $P = 0.791$, Cohen's $d = -0.17$ (95% CI: -1.63 to 1.29). NS, not significant.

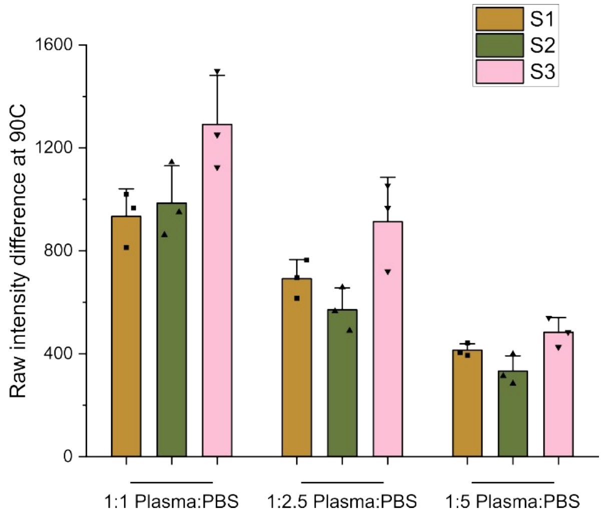

Figure S5. Comparison of average raw grayscale intensity difference measured at 90 C for different dilutions of plasma in PBS. Data are means ± s.d. 3 individual healthy donor samples were tested at 3 different dilution levels, with 3 independent trials for each measurement.

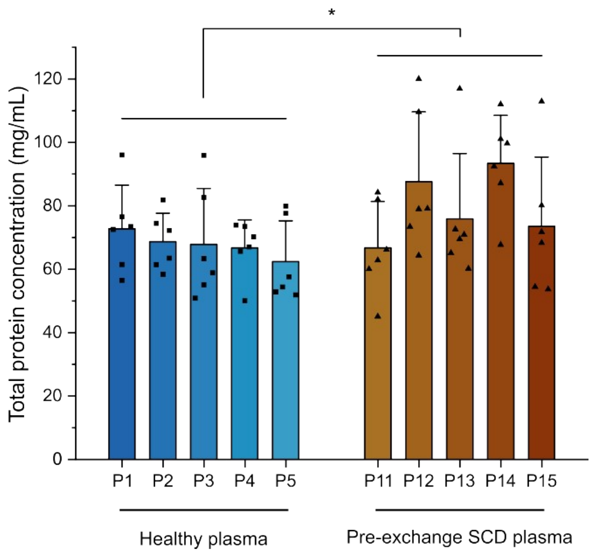

Figure S6. Comparison of total protein concentration measured using BCA assay for individual donors and sorted into healthy and SCD groups. Data are means ± s.d. For each group, 5 individual donors were tested with at least 6 independent trials per donor. Statistical analysis performed on donor-aggregated values using two-sided, unpaired Student's t-test: *P = 0.034, Cohen's d = -1.61 (95% CI: -3.29 to 0.07). *P < 0.05.

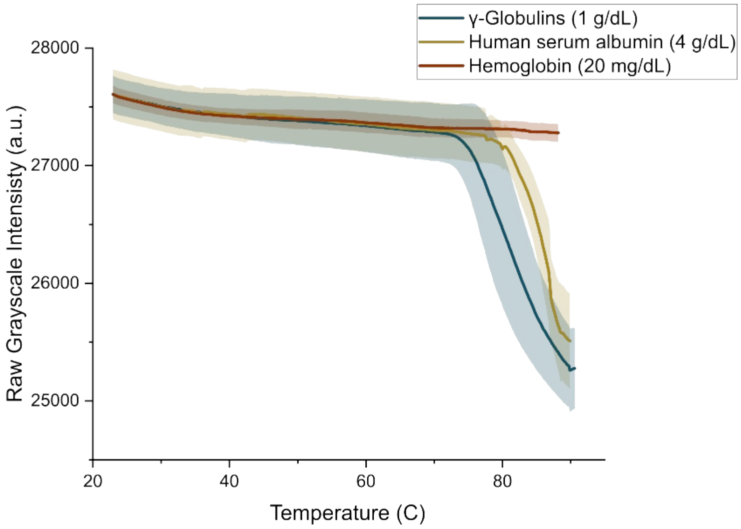

Figure S7. Denaturation curves of various purified proteins found in blood plasma. Samples were tested at estimated physiological concentration. Data are means ± s.d. At least 3 independent trials were conducted for each condition.

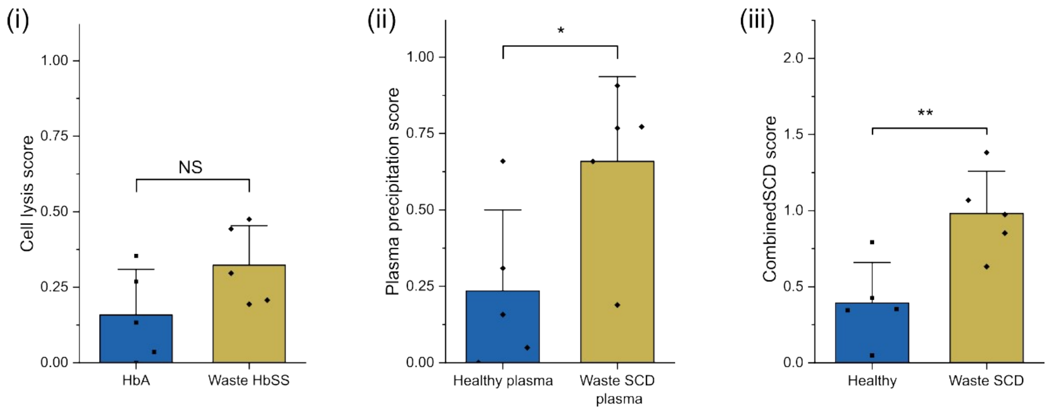

Figure S8. Combined diagnostic comparing healthy donors and post-exchange waste HbSS donors. Normalized score (0-1) for both (i) RBC membrane stability and (ii) plasma protein concentration was generated and added together to produce (iii) combined SCD score (0-2). For each group, 5 individual donors were tested with at least 4 independent trials per donor. Student t-test of independence was performed between individual donors. Cell lysis score: HbA vs. Waste HbSS, P = 0.102, d = -1.17 (95% CI: -2.75 to 0.41), NS; Plasma precipitation score: HbA vs. Waste HbSS, *P = 0.039, d = -1.56 (95% CI: -3.23 to 0.10); Combined score SCD: HbA vs. Waste HbSS, **P = 0.009, d = -2.17 (95% CI: -4.01 to -0.33). **P < 0.01 and *P < 0.05; NS, not significant.

Movie S1 (separate file). SAW application on whole blood sample. Video demonstrates initial dispersed RBCs, immediate patterning when SAW is turned on, eventual cell lysis, and finally protein aggregation as temperature in the channel increases. 5 fps, 4x playback.

Movie S2 (separate file). SAW application on plasma sample. Video demonstrates gradual darkening corresponding to increased aggregation of proteins as temperature in the channel increases. 5 fps, 4x playback.
